# Direct Binding of Cisplatin to p22phox, an Endoplasmic Reticulum (ER) Membrane Protein, Contributes to Cisplatin Resistance in Oral Squamous Cell Carcinoma (OSCC) Cells

**DOI:** 10.3390/molecules25173815

**Published:** 2020-08-21

**Authors:** Chih-Chang Hung, Fu-An Li, Shih-Shin Liang, Ling-Feng Wang, I-Ling Lin, Chien-Chih Chiu, Chiu-Hsien Lee, Jeff Yi-Fu Chen

**Affiliations:** 1 Department of Biotechnology, Kaohsiung Medical University, Kaohsiung 807, Taiwan; hucch0510@gmail.com (C.-C.H.); liang0615@kmu.edu.tw (S.-S.L.); cchiu@kmu.edu.tw (C.-C.C.); 2 Institute of Biomedical Sciences, Academia Sinica, Taipei 100, Taiwan; falee@ibms.sinica.edu.tw; 3 Institute of Biomedical Science, National Sun Yat-sen University, Kaohsiung 807, Taiwan; 4 Department of Medical Research, Kaohsiung Medical University Hospital, Kaohsiung 807, Taiwan; 5 Department of Otolaryngology, College of Medicine, Kaohsiung Medical University, Kaohsiung 807, Taiwan; lifewang@kmu.edu.tw; 6 Department of Otolaryngology, Kaohsiung Municipal Ta-Tung Hospital, Kaohsiung 807, Taiwan; 7 Department of Medical Laboratory Science and Biotechnology, College of Health Sciences, Kaohsiung Medical University, Kaohsiung 807, Taiwan; linili@kmu.edu.tw; 8 Department of Laboratory Medicine, Kaohsiung Medical University Hospital, Kaohsiung 807, Taiwan; 9 National Yujing Senior Vocational School of Technology and Commerce, Tainan 714, Taiwan; lilylee0710@gmail.com; 10 Center for Cancer Research, Kaohsiung Medical University, Kaohsiung 807, Taiwan

**Keywords:** p22phox, cisplatin, drug-protein interaction, chemoresistance, oral squamous cell carcinoma (OSCC)

## Abstract

Prolonged treatment with cisplatin (CDDP) frequently develops chemoresistance. We have previously shown that p22phox, an endoplasmic reticulum (ER) membrane protein, confers CDDP resistance by blocking CDDP nuclear entry in oral squamous cell carcinoma (OSCC) cells; however, the underlying mechanism remains unresolved. Using a fluorescent dye-labeled CDDP, here we show that CDDP can bind to p22phox in both cell-based and cell-free contexts. Subsequent detection of CDDP-peptide interaction by the Tris-Tricine-based electrophoresis revealed that GA-30, a synthetic peptide matching a region of the cytosolic domain of p22phox, could interact with CDDP. These results were further confirmed by liquid chromatography–mass spectrometry (LC–MS) analysis, from which MA-11, an 11-amino acid subdomain of the GA-30 domain, could largely account for the interaction. Amino acid substitutions at Cys50, Met65 and Met73, but not His72, significantly impaired the binding between CDDP and the GA-30 domain, thereby suggesting the potential CDDP-binding residues in p22phox protein. Consistently, the p22phox point mutations at Cys50, Met65 and Met73, but not His72, resensitized OSCC cells to CDDP-induced cytotoxicity and apoptosis. Finally, p22phox might have binding specificity for the platinum drugs, including CDDP, carboplatin and oxaliplatin. Together, we have not only identified p22phox as a novel CDDP-binding protein, but further highlighted the importance of such a drug-protein interaction in drug resistance.

## 1. Introduction

Cisplatin (cis-diaminedichloroplatinum (II) or CDDP), the platinum chemotherapeutic agent, has been one of the most important anticancer drugs for treating solid tumors for decades. To date, CDDP-based chemotherapy, frequently in combination with other chemotherapeutic agents, remains the first-line treatment for head and neck cancers, including oral squamous cell carcinoma (OSCC) [1]. For example, CDDP combined with 5-fluorouracil (5-FU) potentiated the induction of apoptosis in oral cancer cells [2] and gave improved survival of patients with advanced OSCC [3]. Furthermore, the combined treatment of CDDP and epidermal growth factor receptor (EGFR) inhibitors showed enhanced susceptibility to CDDP-mediated apoptosis in OSCC cells [4,5]. However, resistance of cancer cells to CDDP after repeated treatments is the major limitation of the CDDP-based chemotherapy. Cancer cells may lose their sensitivity to the cytotoxic effect of CDDP due to a wide spectrum of genetic or epigenetic alterations. These alterations may (a) affect the cellular processes that occur prior to the binding of CDDP to its targets (pre-target resistance); (b) promote DNA repair or tolerance to DNA lesions (adducts) caused by CDDP (on-target resistance); (c) impair the lethal signaling pathways (e.g., apoptosis) elicited by CDDP-mediated DNA damage (post-target resistance) or (d) activate prosurvival molecular circuitries that are not closely associated with CDDP-elicited signals (off-target resistance) [6].

CDDP is a well-known DNA-damaging agent that causes cytotoxicity in cancer cells. It is generally believed that CDDP-induced cytotoxicity is mediated through its ability to cross-link DNA to form DNA adducts, ultimately leading to the induction of apoptosis if the damages exceed the capacity of DNA repair. Interestingly, there are reports that CDDP also has the ability to platinate and cross-link proteins. More than 50% of the platinum (Pt) from CDDP of pharmacologically relevant doses added to human blood plasma is bound to albumin [7]. Additionally, CDDP has been shown to interact with several cellular proteins, such as copper transporter receptor 1 (CTR1), glucose-regulated protein 94 (GRP94), cytochrome c (cyt c) and heat shock protein 90 (HSP90) [8,9,10]. It is possible that such drug-protein interactions are associated with the toxic side effects or the chemosensitivity of CDDP treatment.

We had previously demonstrated that p22phox, an endoplasmic reticulum (ER) membrane protein essential for the activity of the Nox family NADPH oxidases, conferred OSCC resistance to CDDP in both cell and mouse models [11,12]. Whereas p22phox appeared to block the nuclear entry of CDDP by sequestering CDDP in the cytoplasm [11], the underlying mechanism is not fully understood. In this study, we investigated whether p22phox is also a CDDP-binding protein and may contribute to the resistance to CDDP by this drug-protein interaction in OSCC cells. Our results suggest that p22phox may interact with and thus sequester CDDP in the cytoplasm, compromising the anticancer activity of CDDP in OSCC cells.

## 2. Results

### 2.1. Cell-Free and Cell-Based CDDP Binding to p22phox Protein

**Figure 1:**
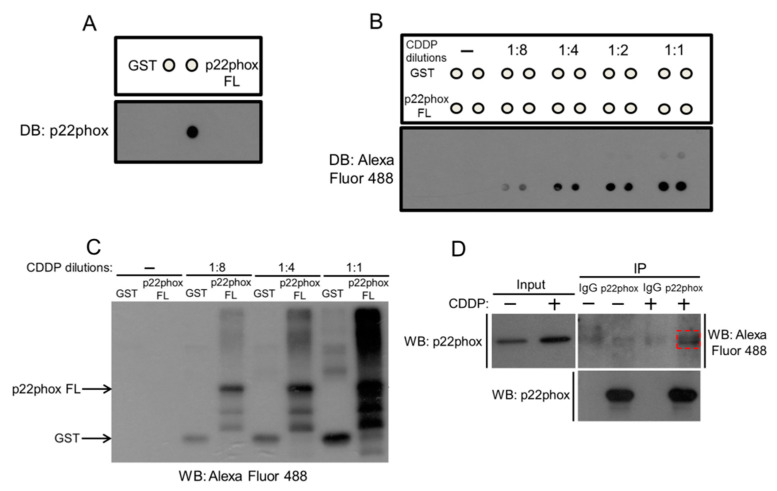
p22phox protein interacts with CDDP in vitro. (**A**) The identity of GST-p22phox full-length recombinant protein (or p22phox FL) was verified by dot blot analysis using anti-p22phox. The recombinant GST and p22phox FL proteins were immobilized to the sepharose GSH beads, and then incubated in PBST buffer with or without increasing dilutions (1:2–1:8) of Alexa Fluor 488-labeled CDDP at 37 °C for 1 h. The beads were analyzed by dot blotting (**B**) and immunoblotting (**C**). (**D**) p22phox-expressing SAS cells were treated with Alexa Fluor 488-labeled CDDP overnight and the cell lysates were immunoprecipitated with anti-p22phox or IgG control, followed by Western blotting with anti-Alexa Fluor 488 (dotted box) or anti-p22phox.

We had previously shown that p22phox protein was almost perfectly co-localized with CDDP in p22phox-expressing OSCC cells [11], motivating us to speculate that p22phox might interact with CDDP. We investigated whether CDDP could bind to glutathione S-transferase (GST)-p22phox full-length recombinant protein (or p22phox FL) in vitro. After verifying the identity of the purified p22phox FL protein (Figure 1A), we showed that CDDP could bind to the recombinant protein by GST pull-down binding assay. More importantly, CDDP had significantly higher binding affinity to the p22phox recombinant protein than to the GST protein control at various incubation dilutions, further suggesting the binding specificity between CDDP and p22phox protein (Figure 1B,C). To support the cell-free binding results, we tested CDDP-p22phox binding in the cell-based context. The result indicated that CDDP could bind to p22phox protein in p22phox-expressing OSCC SAS cells by co-immunoprecipitation (Figure 1D).

### 2.2. Mapping of the CDDP-Binding Domain in p22phox Protein

**Figure 2:**
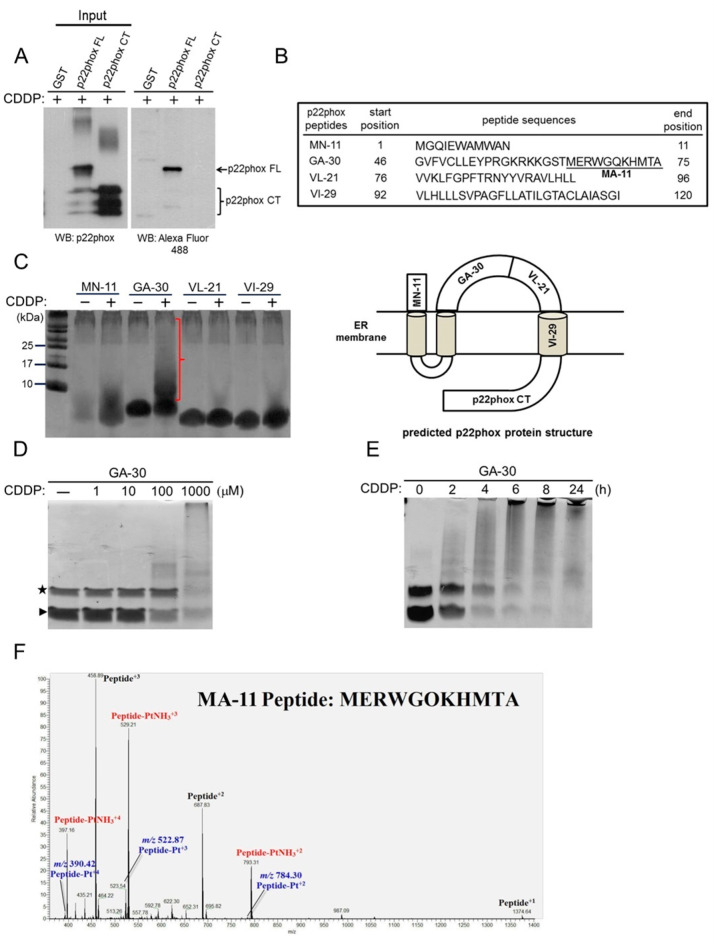
Identification of the CDDP-binding domain in the p22phox protein. (**A**) Binding between p22phox C-terminal domain (p22phox CT) and CDDP was determined using the GST pull-down assay as in Figure 1C. (**B**) (upper panel) The commercially synthesized p22phox peptides and their sequences. (lower panel) The predicted p22phox protein structure and the corresponding locations of the peptides. (**C**) The p22phox peptides, MN-11 (1.3 kDa), GA-30 (3.5 kDa), VL-21 (2.5 kDa) and VI-29 (2.85 kDa), were incubated in PBS buffer with or without CDDP (1 mM) at room temperature overnight. The reactions were analyzed by Tris-Tricine SDS-PAGE and silver staining. (**D**) The GA-30 peptide (100 μM) was incubated with the indicated doses of CDDP at room temperature overnight and then analyzed by Tris-Tricine SDS-PAGE and Coomassie blue staining. The star and arrowhead denote the putative peptide dimer and unbound peptides, respectively. (**E**) The GA-30 peptide (10 μg) was incubated with 1 mM CDDP for the indicated time periods and then analyzed as in (D). (**F**) The LC–MS extracted spectrum of CDDP-modified peptides shown in different charge statuses. The unmodified peptides: *m/z* = 1374.64 with 1 charge, *m/z* = 687.83 with 2 charges and *m/z* = 458.89 with 3 charges. The Pt-modified peptides: *m/z* = 784.30 with 2 charges, *m/z* = 522.87 with 3 charges and *m/z* = 390.42 with 4 charges. The PtNH_3_-modified peptides: *m/z* = 793.31 with 2 charges, *m/z* = 529.21 with 3 charges and *m/z* = 397.16 with 4 charges.

To further dissect which part of the p22phox protein may interact with CDDP, we first generated a p22phox C-terminal recombinant protein (GST-p22phox CT; a.a. 132–195) corresponding to a region of the p22phox ER-luminal domain as predicted by computational modeling (Figure 2B, bottom panel) [13]. GST pull-down followed by Western blotting clearly demonstrated that CDDP could bind to the full-length but not the C-terminal version of p22phox protein (Figure 2A, right panel).

To map the possible CDDP-binding domains, we commercially synthesized four different peptide fragments that collectively cover the entire cytoplasmic domain (MN-11, GA-30 and VL-21) and one of the transmembrane domains (VI-29) of p22phox protein (Figure 2B, upper panel). Using Tris-Tricine SDS-PAGE, we showed that the incubation of the GA-30 peptide, but not other peptides, with CDDP resulted in a decrease of the unbound peptide concomitant with a significant high-molecular-weight band smearing, indicating that CDDP could effectively bind to and even cross-link the peptide (Figure 2C, the parenthesis). In addition, CDDP could bind to the GA-30 peptide in a time- and dose-dependent manner, further ensuring the binding affinity between these two molecules (Figure 2D,E). Furthermore, MA-11, a portion of the GA-30 peptide predictably harboring the “hot-spot” CDDP-binding residues (Met and His) [14,15], was found to be bound to the Pt of CDDP by analyzing the mass-to-charge (*m*/*z*) values in the liquid chromatography-mass spectrometry (LC–MS) spectrum (Figure 2F). Together, these data have conclusively shown the ability of CDDP to platinate the GA-30 domain, possibly leading to the formation of CDDP-p22phox adducts.

### 2.3. Identification of the Potential p22phox-CDDP Interaction Sites in the GA-30 Domain 

**Figure 3:**
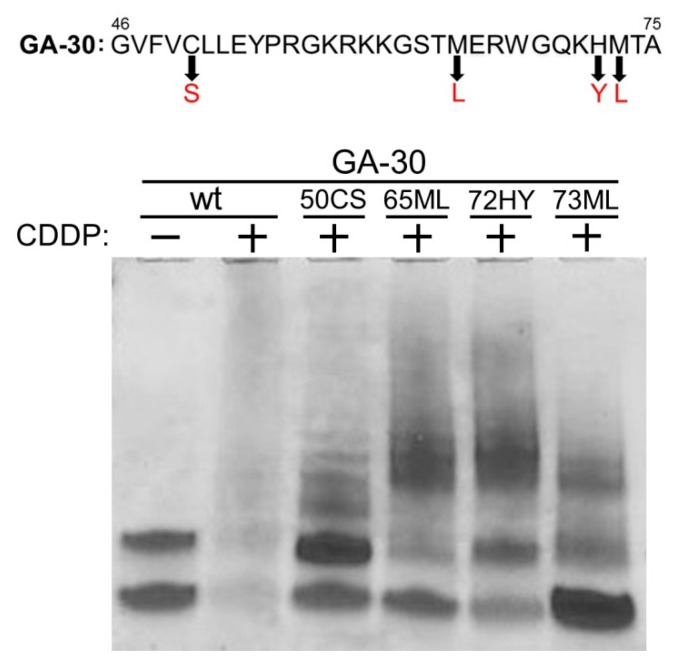
Binding of CDDP to four amino acid residues in the GA-30 domain. The four commercially synthesized GA-30 peptides individually with single amino acid substitution, 50CS (Cys → Ser), 65ML (Met → Leu), 72HY (His → Tyr) and 73ML (Met → Leu), were tested for binding to CDDP. Ten micrograms of the wild type and the mutant GA-30 peptides were separately incubated with 1 mM CDDP at room temperature overnight. The reactions were analyzed by Tris-Tricine SDS-PAGE and Coomassie blue staining.

Based on the chemical properties and previous reports, we speculated that CDDP might bind to four specific amino acid residues, Cys50, Met65, His72 and Met73, in the GA-30 domain of the p22phox protein. We commercially synthesized four GA-30 peptide fragments, each of which harbors the substitution of the four amino acid residues, respectively; namely 50CS (Cys to Ser), 65ML (Met to Leu), 72HY (His to Tyr) and 73ML (Met to Leu). We then tested whether such changes would impact the interaction between GA-30 and CDDP by the Tris-Tricine electrophoresis system. While, as in Figure 2C, the incubation of the wild-type GA-30 peptide with CDDP resulted in effective binding between the two molecules, the amino acid substitutions led to different degrees of incomplete binding; for example, 73ML had the highest amount of remaining unbound peptides in the presence of CDDP, indicating that this amino acid substitution made the most negative impact on CDDP-GA-30 interaction (Figure 3). Thus, each of the four amino acid substitutions could at least partially abolish the overall interaction between CDDP and the GA-30 peptide, suggesting the potential CDDP-binding residues in the p22phox protein.

### 2.4. Disruption of the CDDP-p22phox Binding Sites in the GA-30 Domain Resensitizes OSCC Cells to CDDP-Induced Cytotoxicity and Apoptosis 

**Figure 4:**
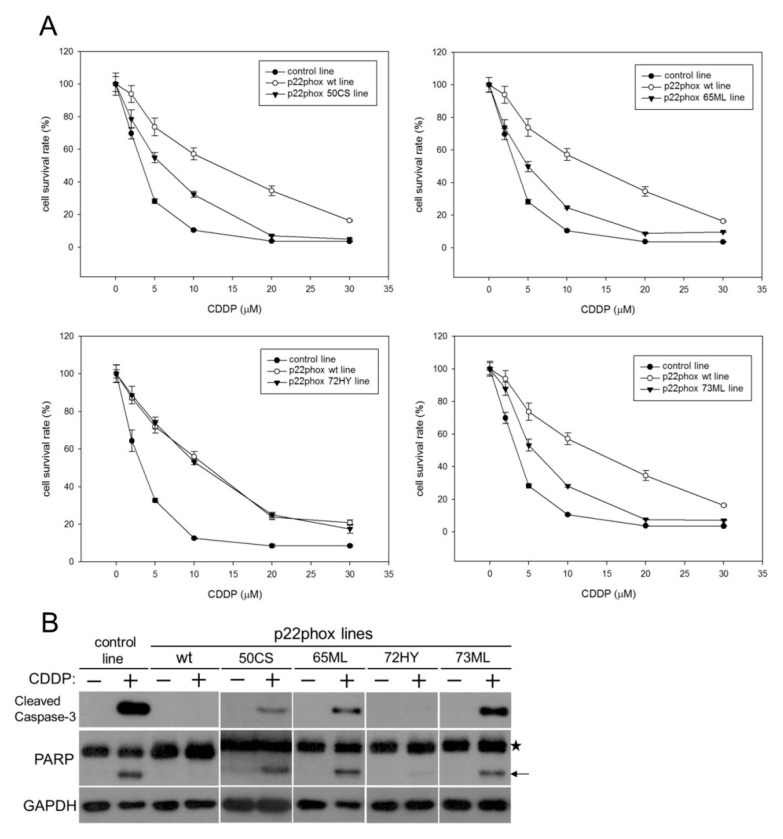
The mutant p22phox proteins alleviate CDDP resistance and recover CDDP-induced apoptosis. (**A**) OSCC SAS cells stably expressing site-directed mutagenized p22phox proteins (50CS, 65ML, 72HY and 73ML) were treated with increasing concentrations (2, 5, 20 and 30 μM) of CDDP for 48 h. Cell survival was determined by the MTT assay. The control line was the parental SAS cells transfected with the empty pDsRed-N1 vector. The survival rate of each stable line without treatment was deliberately set to 100%. All measurements were performed in triplicate and expressed as mean ± SD. (**B**) The cells were treated with CDDP (10 μM) for 24 h and the expression of cleaved caspase-3 (17 kDa) and PARP (89 kDa) was revealed by Western blot analysis. The star and arrow indicate the pro-form and the cleaved form of PARP, respectively.

To understand how the potential CDDP-p22phox binding sites would impact the biological function of p22phox, we generated OSCC cells stably overexpressing the mutant versions of p22phox, i.e., 50CS, 65ML, 72HY and 73ML. We then analyzed the survival rate of the p22phox mutant lines treated with increasing concentrations of CDDP for 48 h. The results showed that p22phox lines carrying 50CS, 65ML and 73ML but not 72HY mutations had significantly reduced survival compared to the wild-type line (p22phox wt line; Figure 4A). Since CDDP is known to kill cancer cells by inducing apoptosis [16], we next confirmed whether the amino acid substitutions in the GA-30 domain would affect CDDP-induced apoptosis. Consistently, CDDP-induced apoptosis was significantly recovered in the 50CS, 65ML and 73ML but not 72HY mutant lines, as evidenced by the markedly increased expression of cleaved caspase-3 and poly (ADP-ribose) polymerase (PARP) compared to the wild-type line (Figure 4B). Together, disrupting the potential CDDP-binding sites within the GA-30 domain at Cys50, Met65 and Met73 of p22phox protein resensitizes the cells to CDDP-induced cytotoxicity and apoptosis.

### 2.5. p22phox Protein Exhibits Binding Specificity to the Platinum Drugs

**Figure 5:**
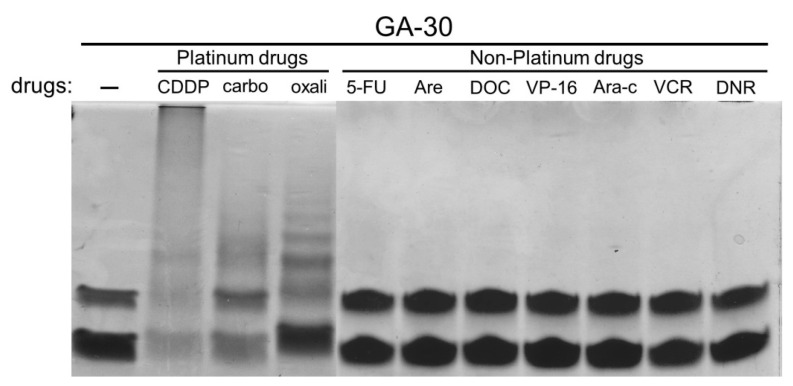
The binding affinity of the GA-30 domain to other chemotherapy agents. Ten micrograms of the GA-30 peptide was individually incubated with 1 mM CDDP, 1 mM carboplatin (carbo), 1 mM oxaliplatin (oxali), 1 mM 5-fluorouracil (5-FU), 0.5 mM arecoline (Are, the irrelevant control), 1 mM docetaxel (DOC), 1 mM etoposide (VP-16), 0.5 mM cytarabine (Ara-c), 1 mM vincristine (VCR) and 0.5 mM daunorubicin (DNR) at room temperature overnight. The reactions were analyzed by Tris-Tricine SDS-PAGE and Coomassie blue staining.

To understand whether the GA-30 domain, which contains the putative CDDP-binding sites, could also interact with other chemotherapy drugs, we performed a “zoo blot”-type binding assay. While the incubation of the GA-30 peptide respectively with the three platinum drugs, including CDDP, carboplatin and oxaliplatin, resulted in the typical band-shift electrophoretic pattern as in Figure 2, the pattern of all six non-platinum drugs, including 5-fluorouracil (5-FU), docetaxel (DOC), etoposide (VP-16), cytarabine (Ara-c), vincristine (VCR) and daunorubicin (DNR), was identical to that of the unbound state (first lane), suggesting that p22phox protein may preferentially interact with the platinum drugs, albeit to different degrees, but not the non-platinum drugs (Figure 5). 

## 3. Discussion

The major problem for the platinum-based chemotherapy is the development of drug resistance that frequently leads to treatment failure. We have previously shown that p22phox confers CDDP resistance by blocking CDDP nuclear entry in OSCC cells; however, the underlying mechanism remains unresolved. The observation that CDDP was nearly perfectly co-localized with the p22phox protein at the nuclear periphery in the cells prompted us to speculate that these two molecules could interact with each other [11]. Indeed, this was verified by the result that CDDP could be co-immunoprecipitated with the p22phox protein in OSCC SAS cells. Furthermore, we showed that CDDP could bind to GST-p22phox recombinant protein presumably with high affinity and specificity. Although CDDP also displayed minor binding affinity to the GST protein control (Figure 1B,C), our results still suggest that it was the p22phox moiety, but not the GST moiety, that contributed primarily to the markedly stronger binding of CDDP to the GST-p22phox fusion protein.

We next investigated to which part of the p22phox protein CDDP may bind. Based on the p22phox protein structure predicted by in silico computational modeling [13], three peptide fragments that collectively cover the entire cytosolic domain were synthesized. Since CDDP becomes an aquated species once entering the cells [17,18], we thought it highly unlikely that such a molecule would further diffuse across the hydrophobic ER membrane into the ER lumen, thereby minimizing the possibility of interaction between CDDP and the transmembrane and luminal domains of the p22phox protein in the cells. Still, we tested the three cytosolic peptide fragments, alongside a recombinant protein and a peptide fragment corresponding to the C-terminal region in the luminal domain (p22phox CT) and one of the transmembrane domains (VI-29), respectively, for binding to CDDP. Taking advantage of the Tris-Tricine gel electrophoresis that can effectively detect and separate small peptide molecules, we demonstrated that CDDP had the strongest binding affinity to the cytosolic GA-30 peptide fragment; in fact, CDDP could even cross-link the peptide fragment based on the significant band shift to high molecular mass (Figure 2C). Moreover, there was virtually no interaction between CDDP and both p22phox CT and VI-29; VI-29 represents the lone transmembrane domain with one hot-spot binding residue, histidine [15]. The dose- and time-dependent binding kinetics between CDDP and GA-30 were further verified. Furthermore, to confirm the binding assay results, a subdomain of GA-30, MA-11, which harbors three binding hot spots, was proven to be bound to CDDP by mass spectrometry. Together, these results suggest that CDDP may bind to the p22phox protein specifically through the cytosolic GA-30 domain. However, the exact CDDP-binding residues in the GA-30 domain remain uncertain. We are currently identifying the binding residues by manually analyzing any possible mass shifts of fragment ions in the second MS spectrum.

To identify the potential CDDP-p22phox interaction sites residing within the GA-30 domain, we focused on the three known hot-spot amino acids, Cys, Met and His [14,19]. Whereas the addition of CDDP to the GA-30 peptide resulted in a substantial peptide adduct formation, amino acid substitutions at Cys50 (50CS), Met65 (65ML), His72 (72HY) and Met73 (73ML) within the peptide differentially diminished the binding of the two molecules. Although these results indicate that CDDP could potentially bind to all the four amino acid residues, there may exist binding selectivity of CDDP toward the four sites, thus contributing differently to the overall interaction between CDDP and the GA-30 peptide. Additionally, since three of the potential CDDP-binding residues, Met 65, His72 and Met73, are located in the MA-11 subdomain, these findings have further confirmed the mass spectrometry results (Figure 2F).

We then determined whether the four potential CDDP-binding residues may impact p22phox-dependent resistance to CDDP in OSCC cells; if CDDP binding to p22phox is crucial for the drug resistance, mutations of the binding sites on p22phox should reverse such an effect. Indeed, we found that OSCC cells carrying 50CS, 65ML and 73ML, but not 72HY, site-directed point mutations exhibited significantly reduced survival rate with CDDP treatment. Moreover, the active forms of caspase-3 and PARP were significantly recovered in the three CDDP-sensitive p22phox mutant lines. These results suggest that disruption of CDDP-p22phox binding through the GA-30 domain resensitizes the cells to CDDP-induced cytotoxicity and apoptosis. However, it is not known whether removal of all the four CDDP-binding sites can further enhance the sensitivity of OSCC cells to CDDP, because overexpression of a GA-30-deleted p22phox protein seemed to be sublethal to the cells (data not shown). On the other hand, the point mutation at His72 (72HY) appeared to have the least impact on CDDP-induced cytotoxicity and apoptosis, consistent with the result that this amino acid residue had the least contribution to the overall interaction between CDDP and the GA-30 domain (Figure 3). While His72 represents a polymorphic site (C242T) of p22phox known to be associated with the risk of cardiovascular diseases [20,21], its role in drug resistance has yet to be reported.

Finally, to explore the possibility that p22phox may interact with other small-molecule anticancer drugs, we tested the ability of several platinum and non-platinum agents to bind to the p22phox protein. Our results showed that, whereas the GA-30 peptide could be evidently bound to the three platinum drugs including CDDP, surprisingly, all the six non-platinum drugs were completely unable to bind to the peptide. These findings suggest that p22phox may preferentially interact with the platinum but not the non-platinum drugs. Notably, based on the remaining amount of the unbound peptides, it appeared to be oxaliplatin < carboplatin < CDDP in that order of increasing binding affinity to the GA-30 peptide. This is in agreement with our previous results that p22phox confers the same order of increasing resistance to the three platinum drugs [12].

## 4. Materials and Methods 

### 4.1. Establishment and Culture of p22phox Wild-Type and Mutant Stable Lines

The parental OSCC cell line, SAS, was obtained and maintained as previously mentioned [11]. SAS cell line was originally derived from a human tongue primary lesion. It is virus-free and has a doubling time 21 h (Japanese Collection of Research Bioresources; JCRB). In addition, the cell line shows hypertriploidy [22] but was reported to be microsatellite stable (the Catalogue of Somatic Mutations in Cancer; COSMIC). Stable p22phox-overexpressing cells, p22phox wt line and the control line were established in our previous studies [11]. Single amino acid substitutions in p22phox were generated by QuikChange site-directed mutagenesis kit (Agilent, Santa Clara, CA, USA). Briefly, a construct carrying wild-type DsRed-p22phox was used as the parental plasmid. The site-directed mutagenesis PCR products were treated with the restriction enzyme DpnI and then transformed into bacteria for amplification and confirmation by DNA sequencing. Establishment of p22phox mutant stable lines was done by transfecting SAS cells with the mutant constructs (20 μg) and selected with medium containing 2 mg/mL G418 for about 4–6 weeks [11]. These cells were routinely maintained in Dulbecco’s modified Eagle medium supplemented with nutrient mixture F-12 (DMEM/F12; GIBCO, Invitrogen Corporation, Waltham, MA, USA) and with 10% FBS.

### 4.2. Production of GST-p22phox Recombinant Proteins 

p22phox full-length (FL) or C-terminal (CT) coding sequence (CDS) was subcloned from the DsRed-p22phox construct into the pGEX-4T-1 vector (GE healthcare, Danderyd, Sweden), and the resulting constructs, GST-p22phox FL and GST-p22phox CT, were transformed into the *E. coli* *Stbl3* strain (Thermo Fisher Scientific, Waltham, MA, USA). After growing the bacterial cultures overnight at 37 °C, recombinant protein expression was induced by the addition of 0.2 mM IPTG to the culture at room temperature overnight. The bacterial cells were collected by centrifugation at 4 °C and resuspended in 10 mL PBST (1% Triton X-100) buffer. Bacterial lysates were obtained by sonication on ice and then removal of insolubl

e pellets by centrifugation. To purify the recombinant proteins, 1 mL of glutathione (GSH) sepharose (GE healthcare, Danderyd, Sweden) was added into the bacterial lysates, and the GSH sepharose mixture was incubated overnight at 4 °C. The sepharose beads were washed in the PBST buffer, and the GST-tagged recombinant proteins were eluted by fresh 10 mM reduced GSH (Sigma, St. Louis, MO, USA). The resulting GST-p22phox FL and GST-p22phox CT proteins were verified by Western blot analysis.

### 4.3. CDDP-p22phox Recombinant Protein Binding Assay

Binding of CDDP to p22phox recombinant proteins was validated by GST pull-down-based binding assay. Briefly, GST, GST-p22phox FL or GST-p22phox CT protein (16 μg each) was immobilized to the GSH sepharose beads at 4 °C for 1 h. The protein-bound beads were incubated in the PBST buffer with or without Alexa Fluor 488-labeled CDDP (Thermo Fisher Scientific, Waltham, MA, USA) at 37 °C for 1 h. After washing the beads with the PBST buffer, the CDDP-bound protein samples were eluted by the SDS sample buffer. The samples were then analyzed by dot blot or Western blot analysis using antibodies against p22phox (Santa Cruz, CA, USA) or Alexa Fluor 488 (Thermo Fisher Scientific, Waltham, MA, USA). For cell-based binding assay, after treated with Alexa Fluor 488-labeled CDDP overnight, the p22phox-expressing SAS cells were lysed in RIPA buffer and cell lysates were collected. The lysates (200 μg) were then incubated with Protein A/G beaded agarose and anti-p22phox antibody (10 μg) at 4 °C overnight. After washing with RIPA buffer, the immunoprecipitates were analyzed by immunoblotting using anti-Alexa Fluor 488 antibody.

### 4.4. CDDP-p22phox Peptide Binding Assay

Binding of CDDP to p22phox wild-type or mutant peptide fragments was verified using the Tris-Tricine electrophoresis system [23]. The peptides were commercially synthesized with more than 85% purity (Kelowna International Scientific Inc., Taipei, Taiwan) and dissolved in PBS buffer. Ten micrograms of each peptide fragment in PBS was incubated with 1 mM CDDP (BioVision, Milpitas, CA, USA) overnight at room temperature. The CDDP-peptide interaction was detected by 16.5% Tris-Tricine gel electrophoresis and then Coomassie blue (or sliver) staining.

### 4.5. Detection of CDDP-Peptide Binding by LC–MS/MS

CDDP-incubated peptides were desalted with C18 Ziptip (Millipore, Burlington, MA, USA) according to the manufacturer’s instructions. The LC–MS/MS analysis was performed on a nanoAcquity UPLC system (Waters) coupled with an Orbitrap Elite mass spectrometer (Thermo Scientific). The peptide samples were separated on BEH C18 column (130 Å, 1.7 µm, 75 µm × 250 mm, Waters) using a gradient from 5% to 35% solvent B in 90 min (solvent B: 0.1% formic acid in acetonitrile) at a flow rate of 300 nL/min. The mass spectrometer was operated in the data-dependent mode with the following acquisition cycle: a full scan (*m*/*z* 350–1600) recorded in the Orbitrap analyzer at resolution R = 240,000, and up to the 10 most intense peaks with charge ≥ 2 were selected and fragmented by collision-induced dissociation (CID) and higher-energy collision dissociation (HCD) with normalized collision energy of 35% and 28% individually. The raw data were processed by Xcalibur software (version 2.2, Thermo Finnigan Inc., San Jose, CA, USA).

### 4.6. Cell Survival Assay

The p22phox wild-type and mutant SAS cells were seeded into 24-well plates at a density of 7 × 10^4^ in each well. The cells were treated with various concentrations of CDDP for 48 h. Cell survival was evaluated by Methylthiazol tetrazolium (MTT) assay (Chemicon International Inc., Temecula, CA, USA) as previously mentioned [11].

### 4.7. Western Blot Analysis

Briefly, total cell extracts (12 μg/sample) were resolved by 10–12% Tris-Glycine SDS-PAGE, followed by the procedures as previously described [11]. The expression of the cleaved caspase-3 and PARP was detected by rabbit anti-cleaved caspase-3 and rabbit anti-PARP antibodies (Cell Signaling, Danvers, MA, USA), respectively.

## 5. Conclusions

To our knowledge, this is the first reported evidence of direct binding between p22phox protein and small-molecule anticancer drugs; on the other hand, we also identified a novel CDDP-binding protein. More noticeably, while a recent study reveals that siRNA knockdown of some CDDP-binding cytosolic proteins sensitizes colon cancer cells to CDDP [24], our findings may further underline the importance of the interaction between CDDP, and possibly the platinum drugs, and their binding protein partners. In conclusion, these results suggest that p22phox confers resistance to CDDP by directly interacting with and thus sequestering CDDP in the cytoplasm in OSCC cells, which provides a new mechanistic insight into CDDP resistance.
